# Integrating Computational Modelling into the Ecosystem of Cochlear Implantation: Advancing Access to Diagnostics, Decision-Making, and Post-Implantation Outcomes on a Global Scale

**DOI:** 10.3390/jcm14227929

**Published:** 2025-11-08

**Authors:** Tania Hanekom

**Affiliations:** Bioengineering, Department of Electrical, Electronic and Computer Engineering, University of Pretoria, Pretoria 0002, South Africa; tania.hanekom@up.ac.za

**Keywords:** cochlear implant, biophysical model, model-facilitated care, clinical implementation, computational biophysiology

## Abstract

Disabling hearing loss affects more than 5% of the global population, with numbers expected to double by 2050. The burden is especially high in low- and middle-income countries, where access to cochlear implant (CI) technology and the required follow-up care is limited. While CIs are a proven treatment for certain types of hearing loss, their adoption in these countries is hindered by high costs, the need for specialised rehabilitation, and the financial and time commitment required for long-term device maintenance. Although remote programming has improved accessibility to standard care, specialised interventions for complications remain restricted mainly to areas with clinical centres. Computational modelling offers a promising solution to this access-to-care dilemma. The models may be used to simulate complications, such as non-auditory stimulation (NAS), to investigate and plan personalised interventions, and ultimately predict device parameters, without requiring the recipient’s physical presence. Both phenomenological and biophysical models have already demonstrated useful application in CIs: the former streamlines clinical workflows and aims to establish consistency in device fitting, and the latter provides insights into patient-specific auditory biophysiology. Despite decades of research, clinical translation of biophysical models has been limited by data constraints, parameter uncertainty, and validation challenges. In this perspective piece, it is argued that biophysical models have now reached sufficient maturity to be integrated into routine CI care. Apart from the advantages that this approach will bring to the overall advancement of person-centred CI care, it is envisioned to improve accessibility, personalisation, and long-term outcomes for CI recipients in low- and middle-income countries.

## 1. Introduction

The World Health Organisation (WHO) estimates that more than 5% of people suffer from disabling hearing loss. This number is projected to grow to about 10% by 2050, amounting to over 700 million people who will have to live with this disability. An estimated eighty per cent of people with disabling hearing loss live in low- and middle-income countries (LMICs) [1,2,3]. Disabling hearing loss affects a person’s ability to communicate with others and may affect psychosocial well-being [2,4]. Furthermore, children with hearing loss often do not receive proper schooling or perform worse than their hearing peers [3], while adults with hearing loss typically have a higher unemployment rate than their hearing counterparts [3,4].

Cochlear implant (CI) technology is a proven treatment option for hearing impairment [5]. However, the devices are expensive due to the sophisticated technology and rigorous testing required to bring implantable medical devices onto the market, as well as sustaining the business operations of the manufacturing companies. This is particularly relevant in resource-constrained regions, such as Sub-Saharan Africa. Furthermore, even if initial funding to receive a cochlear implant can be secured, maintaining the device requires a lifelong commitment of time and financial resources [6,7]. It requires extensive auditory training and speech therapy during the rehabilitation process [8,9]; continual visits and access to CI clinical services to maintain the user’s hearing, which may change over time as a result of ongoing physiological processes [9]; expert intervention should complications, such as NAS, arise [8]; device maintenance in the form of replacement of batteries and worn parts; and periodic upgrades as the technology evolves with time and improvements become available to enhance the user’s experience. A recent study investigated factors that affect post-operative experiences in adult CI users. The authors found five main themes: financial considerations, complications, device usability and durability, device programming and adaptation, and patient motivation and support [10]. These closely align with the investment required to reach a functional level of use and maintain the CI over the user’s lifetime.

## 2. Cochlear Implants Are Not Generally Accessible

Cochlear implant technology has not yet been widely adopted in developing countries, primarily because of the cost and access to care [2,7]. For those fortunate enough to receive an implant, several factors affect access to essential specialised continuing care options, especially in low-income and remote areas. A particular challenge is the practical and financial burden that travelling poses to CI recipients in remote areas. Although the feasibility of remote programming of CI devices has been established [11], access to novel and highly specialised developments in the care and maintenance of CI users is often limited to those living and working in the direct vicinity of the academic or private centres that drive these initiatives [2,8]. Apart from the issue of access to clinical professionals and centres potentially affecting candidacy for large numbers of hearing-impaired individuals in LMICs, the availability of care beyond standard practice becomes very important when complications arise, such as NAS, for which diagnostics and intervention are required.

## 3. Computational Models as a Solution?

**Figure 1:**
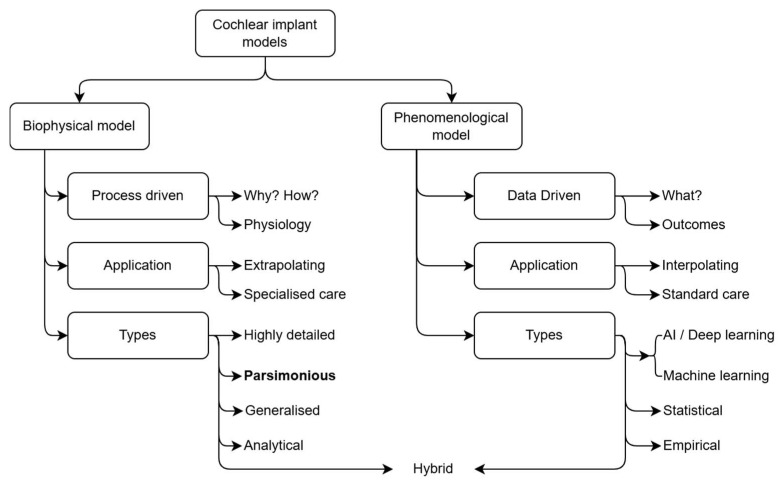
The cochlear implant model space can be divided into biophysical models that aim to describe the anatomy and physiological processes, and phenomenological models that link input to output without regard for the processes that define the relationship. While phenomenological models describe what phenomena occur by describing the transformation from input to output, biophysical models seek to uncover why and how they function, elucidating the underlying processes, interactions, and mechanisms that generate observable behaviour. This means that biophysical models can extrapolate based on a fundamental description of the underlying processes, while phenomenological models perform better when interpolating based on the input-output relationships captured in the description of the model. The complexity of biophysical models and with it the approach to their construction (type) relates to the level of detail captured in the model, ranging from simple analytical models to highly detailed models requiring numerical methods to solve them. The type of approach used to implement a phenomenological model depends on the complexity of the input-output relationship and ranges from empirical models at the lower end of complexity to AI and deep learning models that are suitable to capture highly complex and nonlinear input-output relationships. Hybrid models combine biophysical and phenomenological models, often to create end-to-end models that describe the full auditory pathway [19].

Computational models promise a possible solution to the access-to-care dilemma, as they could potentially be used to predict device programming parameters, explore side effects such as NAS, and assess possible interventions in the physical absence of the CI recipient. Figure 1 summarises the computational CI modelling space. Computational CI models can be broadly categorised into biophysical and phenomenological models, where the former aim to describe and explain the biophysiological processes that underpin the auditory system’s functioning (for acoustic or electric hearing), while the latter aim to capture the relationship between input and output without regard for the underlying processes.

The utility of the two approaches stems from their ability to either extrapolate or interpolate existing data. As the name implies, phenomenological models describe phenomena; in other words, these data-driven models generalise observations of the relation between cause and effect. For this reason, phenomenological models are useful in general care situations, such as predicting optimal mapping parameters based on typical outcomes. The intelligent artificial intelligence (AI) agent, FOX (Fitting to Outcomes eXpert), which was developed to facilitate CI programming [12,13] and was introduced at least a decade before the machine learning boom of the 2020s [14], is an example of a model with prospective clinical use [13] that optimises mapping parameters based on the user’s audiological performance. Big data models, similar to FOX (AI-FOX: https://otoconsult.com/products/fox/), are being incorporated into clinical software [15], aiming to enhance the implant fitting workflow in terms of the speed at which optimal patient outcomes can be achieved. The use of objective fitting software agents also targets the challenge of variability in outcomes by improving consistency in the mapping workflow.

Hybrid models that combine computational anatomical models in the biophysical domain with machine learning or statistical models in the phenomenological domain to infer obscured morphological details have also found valuable clinical applications. An example is the Otoplan software (https://www.medel.pro/precision-tools/ps/precision-surgery/otoplan) [16,17], which is routinely used in surgical planning, electrode model selection [18], and anatomy-based fitting of implants [16].

It should be noted that while specific software such as Otoplan and FOX are referenced as examples of established use of modelling and prediction tools in CI management workflows, the general approach is vendor-neutral and may equally well apply to innovations in software suites such as MAESTRO, version 11 (Med-EL, Innsbruck, Austria) and Custom Sound Pro (Cochlear Ltd., Sydney, Australia).

The full spectrum of auditory processing in CIs, from the periphery to cognitive processing, is covered by a variety of computational models, each tailored to describe a specific processing stage. End-to-end models combine models of different auditory processing stages to provide a comprehensive description of the entire auditory pathway. For example, the model by Brochier et al. [19] combines a three-dimensional (3D) electroanatomical model of the peripheral auditory system, a physiologically based auditory nerve fibre model, and an automatic speech recognition (ASR) neural network to predict CI speech perception. The input to the electroanatomical model of the cochlea is electrical stimuli on individual electrode contacts as generated by applying a CI processing strategy to recorded speech. The spread of electric potential at the output of this model is applied as input to the auditory nerve model. By combining multiple single-fibre models, the nerve model translates the electric potential distribution to corresponding neural response patterns across fibres, which the ASR system then interprets as speech. It is important to note the application of the neural network to describe the higher-order processing stages that are characterised by complex, nonlinear relationships. Artificial intelligence, therefore, enables coupling data-driven learning with biophysiological auditory modelling. This integration has great potential to address some of the remaining challenges in CI listening, such as speech understanding in demanding listening conditions and complex soundscapes, such as music [20].

Models presently employed in clinical applications, e.g., the FOX AI agent, primarily address standard care issues aimed at streamlining and improving user support and device maintenance. This commitment to streamlining candidacy to support and maintenance workflows is, of course, one of the reasons that CI technology is so successful. Similar technologies, e.g., visual prostheses, have failed to gain commercial success partly because of a lack of continued support [21,22]. However, to model subtle inter-person variability that may be obscured in population averages, i.e., to extrapolate from existing data to the idiosyncratic, it is necessary to recognise the value of biophysical models that capture the intricacies of foundational processes. Person-specificity that extends beyond typical population behaviour needs to be captured and described by models that may extend from the level of tissue impedances, morphological differences in structures such as the spiral lamina and neural survival patterns. The remainder of this article explores how biophysical modelling can be incorporated into the CI management protocol to complement standard care AI models. and support more personalised and adaptive care. 

Creating a digital twin of a CI recipient’s peripheral hearing system through computational modelling is an established method in prosthetic hearing research [23,24,25,26,27]. The models aim to describe the biophysical interface between the auditory system and the device, as well as the neural excitation outputs resulting from electrical stimulation of this system. Four types of these models have emerged over the past decades: highly accurate, person-specific models that are constructed directly from segmentation data extracted from high-resolution imaging data [27,28,29], parsimonious or selectively detailed models, delineating important structures, and derived from morphometric measurements on clinical images that may be of a lower resolution [30,31,32,33], generalised anatomical models that ignore inter-person variance in cochlear morphology [19,34], and analytical models that disregard the anatomy and only focus on the electrical decay of the stimulus from the electrode contacts [35,36].

To be useful in a clinical context, a model must capture inter-person variance [32,33,37,38,39]. This disqualifies the generalised and analytical models that purposefully exclude inter-person variance. Highly detailed segmentation-based models have three primary drawbacks when considering clinical deployment. Firstly, these models require high-resolution image data. This requires state-of-the-art clinical imaging equipment, or micro-computed tomography imaging, which is not suitable for live CI recipients. In resource-limited environments, state-of-the-art imaging is often unavailable, meaning that models may need to be constructed from lower-resolution images. Secondly, constructing these models requires considerable effort to clean the segmentation data so that a solvable mesh [25] can be created that is suitable for numerical analysis. Lastly, highly detailed segmentation-based models are often less manipulable than models containing fewer details, which may complicate adjustments to the models that would allow investigating diagnostic possibilities or potential interventions. These models are therefore mostly employed in research settings where the end-to-end modelling time is not a critical factor. Parsimonious models, where less important anatomical details are traded off for a decrease in end-to-end modelling time, are therefore the most viable approach to generating models within a time frame that would allow deployment in a clinical context.

## 4. The Present Adoption of Biophysical Modelling in Clinical Care Protocols

While much biophysical computational modelling work has been conducted in CIs over the past three and a half decades, a common opinion is that although predictive biophysical models are informative [40], their utility in a clinical environment has yet to be proven. This observation is further substantiated by the lack of published literature on biophysical modelling as part of the standard clinical pre-operative work-up protocol [41] and post-operative care protocol for CI recipients. In other words, the utility of computational CI models has yet to cross the clinical boundary between the present use of computational anatomy, which mostly informs surgery and anatomy-based mapping [16], and computational biophysiology, which could inform potential outcomes, programming parameters, and the maintenance and care of CI recipients on a personal and day-to-day basis.

This is not an uncommon scenario for clinical prediction models, as most do not progress to the implementation stage in healthcare settings [41,42]. The clinical model development pipeline can be broken down into three main steps: model development, internal and external validation, and impact assessment [41,42]. The most pertinent reasons for biophysical CI models not reaching the clinical implementation stage are parameter uncertainty [40] impacting the development phase, and a lack of validation [32].

## 5. Perspective

While most research focused on the development of biophysical CI models to understand the intricacies of the electrically stimulated auditory system and the factors that underlie variations in outcomes, it is proposed that the development of these models has reached a level of maturity that warrants a collective effort to deploy biophysical computational models as an integral part of CI recipient care. While the motivation for such a step may be underpinned by the desire to further personalise CI performance optimisation in countries where medical care is readily available, the impact in developing countries may be far more profound. Recognising that the uptake of CI technology in LMICs is greatly impacted by the unavailability and/or high cost of post-implant care, a strong case can be made for developing technologies that may provide care beyond remote programming of the CI.

Although biophysical CI models may not yet be able to predict absolute outcomes, they already offer the potential to gain a greater understanding of the specific auditory situation of an individual CI recipient through the prediction of trends [43]. There is still much work to be performed for these models to reach levels of accuracy that could dictate clinical decisions, but this is envisioned to develop with time as these models are more commonly employed in clinical settings. The most important factor to translate these models from a research setting to standard clinical application is that the development of the models should occur in a transdisciplinary context where modellers (scientists and engineers), model users (clinicians), and model subjects (CI recipients) and their families, reach a mutual understanding of the objectives of the modelling and the expectations to be met [1].

**Figure 2:**
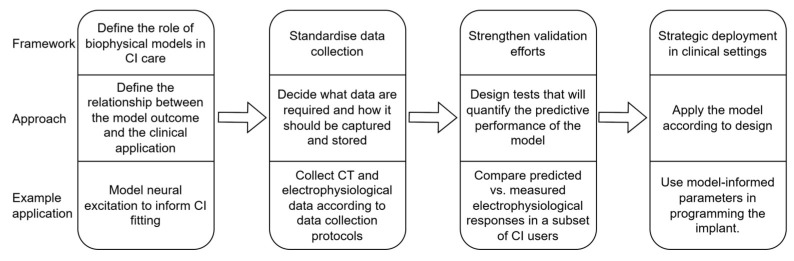
The framework for introducing biophysical cochlear implant models into clinical care protocols is shown in the top row of the diagram. The approach to realising the framework is presented in the middle row, while the bottom row illustrates an example application of the framework, in which a model of neural excitation is developed to inform CI fitting.

Figure 2 shows a proposed framework (top row) for supporting the implementation of biophysical models in standard clinical care protocols. The figure also illustrates the approach for applying the framework (middle row) and an example application of the framework (bottom row). The framework for introducing biophysical models into clinical settings should not be confused with the modelling workflow, which involves data collection, model creation, validation, and application in that order [32,44,45].

### 5.1. Defining and Articulating the Role of Biophysical Models in Clinical Care

Clinicians and modellers must discuss and agree on the contribution that models can make in a clinical setting. The conversation should include what “useful” means and what needs to be improved for the models to be useful.

Examples of useful applications of models include monitoring changes in a CI recipient’s hearing system over time and explaining these changes. For example, bone conductivity can change over time due to disease [31,46], which may affect hearing thresholds and cause NAS, such as facial nerve stimulation (FNS). Likewise, scar tissue formation may affect thresholds over time [47,48]. If a baseline model could be created when a person is implanted, changes in their hearing performance may be monitored, investigated and explained through models. Possible interventions to alleviate complications may also be investigated through modelling. Since these models can be constructed from available imaging data, modelling could be performed in the absence of the CI recipient and thus irrespective of the person’s physical location.

It has also been argued that another useful application for models, which at this point has not yet been fully realised, is the prediction of outcomes [39]. Outcome prediction could contribute to realistic individual expectations of the benefits of implantation and inform additional rehabilitation for CI recipients who underperform. It may also inform individualised electrode designs.

Moreover, models may be employed to predict objective mapping parameters. This mapping may extend beyond anatomy-based fitting (which is primarily based on the length of the cochlear duct) to include knowledge about the state of the nerve and the individual’s unique 3D cochlear morphology.

### 5.2. Purposeful Data Collection

Data collection for modelling must be improved and standardised, especially given that a relatively small number of parameters are consistently collected in clinical settings [49]. Consistent data collection is a crucial requirement for driving present modelling efforts toward clinical adoption. In many cases, data are lost over the course of a CI recipient’s implant life cycle. This presents challenges when complications or changes in performance occur, which could be investigated through models. The situation may be aggravated in LMICs where rigorous data preservation protocols may not have been established. It is therefore proposed that the modelling community reach a consensus on a definition of a minimum dataset required for person-specific biophysical modelling. This consensus dataset requirement must subsequently be communicated to CI centres to establish comprehensive data preservation protocols, if not already in use, and to CI recipients at the candidacy stage to ensure that they share the responsibility for preserving their data. While these datasets may furthermore contribute to big data in CI research, big data may not capture or may filter out person-specific phenomena, highlighting the need to preserve data at a personal level.

A minimum dataset should support the construction and validation of models as well as the assessment of the impact of biophysical modelling in the clinic. To describe the three-dimensional morphology of the cochlea and associated electrical tissue parameters, imaging data, as well as the history and progression of medical conditions that could affect the auditory system, such as meningitis, are required. These data will also be useful in monitoring changes in performance that could be tied to changes in the structure or tissue characteristics of the cochlea and surrounding structures. Programming parameters from the device can offer insights into cochlear biophysiology, for example, by indicating regions with poor neural survival or revealing anomalies such as dehiscences that may trigger FNS and necessitate electrode deactivation. For model validation, device programming parameters, such as thresholds and dynamic ranges (DRs), may again be useful, together with electrophysiological measures, including electrically evoked compound action potentials (ECAPs) and impedance data. Quantitative measures such as speech recognition scores in quiet and noise, comparative analyses on programming parameters such as DR, and psychoacoustic measures such as pitch discrimination may be used to assess the impact of modelling on performance. Likewise, qualitative measures such as listening effort, quality of life and subjective benefit measures may be employed to assess the impact of modelling in clinical settings.

Another important dataset that needs revisiting is the electrical parameters for tissues. Most of these parameters have been measured on animals and date back several decades [43]. Although selected parameters, for example, bone impedance, are often tuned to match measured and predicted quantities such as voltage distributions [32,45,50], a lack of measured data for humans still creates uncertainty in predictions [40].

### 5.3. Validation of Biophysiological Models

Large-scale validation of biophysical models remains a significant challenge, as it requires extensive, high-quality datasets and consistent evaluation across different clinical contexts. Validation requires that model predictions are subjected to the same analyses as the corresponding empirical data, to verify that simulations capture important qualitative and quantitative effects for specific parameter conditions [51]. It is important to realise that validation must extend beyond internal comparisons with datasets that were used to construct a model and should include external validation against independent datasets. Within the context of person-specific models, the implication is that the modelling workflow must firstly be validated by assessing the quality of the models produced for a specific data and parameter set. Once the fidelity of the models produced by the workflow is established, it can be trusted to create accurate models of new datasets. Highly detailed models [27,44] may, for example, serve as ground truth against which parsimonious models may be assessed. Such an assessment would include morphological accuracy and the accuracy of predicted potential distributions resulting from electrical stimulation.

Datasets should ideally span multiple levels of description (e.g., morphological, electrophysiological, psychoacoustic, behavioural, and clinical outcome data) within the same person to support the development of person-specific models. If validation is performed incorrectly, models may yield misleading conclusions about a system, which can have serious consequences if applied in a clinical context [52]. A lack of rigorous validation has been identified as a considerable gap in biophysical modelling of CIs [32,42].

To bridge this gap, it is proposed that a standard validation framework [53] be created for biophysical modelling in CIs. Such a framework must define not only the role of modelling in clinical care (as outlined in Section 5.1) but also the criteria for model performance, including quantitative metrics for predictive accuracy (i.e., quantify how closely an ECAP can be predicted through error metrics such as mean absolute error), thresholds for acceptable error (e.g., predictions of stimulation thresholds should fall within the range of threshold variations on an electrode which is approximately one to two decibels [54]), and requirements for robustness under parameter variability (i.e., the models must remain accurate for variability in electrode placement, cochlear morphology, neural survival patterns and tissue impedances). The validation framework should include sensitivity and uncertainty analyses to ensure that predictions are not artefacts resulting from poor assumptions.

Establishing standardised, open-access validation datasets that can be used for external validation and benchmarking of models, such as the highly detailed models mentioned before, is essential to the validation objective. To be effective, these datasets must be collected using standard protocols to ensure consistency that would allow them to be shared among researchers. Ethical and privacy concerns must also be considered when sharing data. Given the limited availability of invasive human recordings, validation strategies may include alternative data sources, such as animal data or inferred data, e.g., defining approaches to interpret neural excitation as perceptual thresholds [50].

Lastly, it is important that validation frameworks are developed through collaboration among modellers to ensure consensus and a strategy to update and maintain the framework and associated datasets.

### 5.4. Strategic Deployment of Models

Integrating biophysical models into clinical practice for CIs needs a coordinated, transdisciplinary effort between research and clinical teams. Such an initiative requires a well-defined framework to outline how modelling activities are organised, which resources are required, and how these resources should be managed and distributed among participating teams.

Because of the complexity of the models, an iterative development and deployment process is proposed. Initially, two or three collaborating teams should focus on one or two specific model objectives in a limited clinical setting. For example, models could target CI recipients experiencing implant complications, providing insights into causes and potential interventions. As these models are refined through iterative testing and validation, the collaboration can expand to include additional teams, growing toward a global network. This network, comprising cochlear implant teams and a global modelling group, would facilitate the sharing of computational models, tools, and expertise. Moreover, effective clinical adoption will depend on training and continuous support for clinical staff to interpret and apply model outputs appropriately within care pathways.

Data collection is critical to this effort, as outlined in the previous sections. A well-organised, comprehensive shared data pool will support model refinement. For extension into resource-limited settings, data collection may leverage accessible technologies, such as cone-beam computed tomography (CBCT), which may be available through dental practitioners, as well as remote care strategies.

Deployment of computational models in CI care raises important ethical concerns. Firstly, end-users of the models, e.g., clinicians and their patients, need to appreciate uncertainties in predictions, from an understanding of the inherent limitations in the model generation process. The process must thus be transparent to foster trust in the way that the models will be applied in the management and care protocol. Explicit patient consent must be obtained to cover model-assisted decision-making in the care and maintenance protocol. This consent must again be based on an understanding of the limitations of the models’ predictive scope. Lastly, liability boundaries must be defined to protect clinicians and model developers in cases where model-based decisions lead to suboptimal outcomes. Emerging guidelines such as DECIDE-AI [55], which provides a framework to evaluate the safe, ethical, and transparent implementation of AI-based decision-support tools in clinical practice, can be adapted to direct the responsible deployment of computational modelling in cochlear implant management.

## 6. Discussion and Conclusions

Computational models have the potential to enhance CI care by supporting a person-specific, evidence-based approach to engage with and manage a recipient’s auditory needs. Apart from predicting responses to electrical stimulation, the models also provide a means to improve understanding of the auditory system, investigate CI complications and explore interventions. Responsible and ethical use of these models is very important, requiring transparent communication of their capabilities and limitations to all stakeholders [56], i.e., modellers, clinicians, CI recipients and their families. Discussions between stakeholders should focus on whether and how a model’s output can be made helpful in answering important questions, ensuring that expectations and perceptions about the power and utility of models are managed. This is further underscored by recent findings, which highlight the complex psychosocial dynamics experienced by families of children with cochlear implants, including elevated parental stress linked to clinical outcomes [57]. Such evidence reinforces the need for careful communication and shared understanding when integrating computational tools into clinical decision-making.

The organisational model that supports CI care, e.g., the comprehensive South African Cochlear Implant Group (SACIG) clinical guidelines [58], provides a robust framework for the delivery of clinical services. Computational models are not intended to replace such established organisational structures but to complement and strengthen them. Biophysical and predictive modelling can support organisational frameworks by informing patient stratification, optimising clinical workflows, and guiding individualised intervention strategies. Furthermore, the integration of computational tools with telemedicine-based care could enhance accessibility for individuals living far away from implant centres. In this context, strengthening digital infrastructure and ensuring reliable internet connectivity become essential components for translating model-assisted decision-making into equitable clinical practice.

Understanding the integration of biophysical models into clinical care also requires consideration of the broader health context of CI users. Hearing loss frequently co-occurs with a range of systemic comorbidities that have implications for audiological care [59]. These interdependencies underscore the need for future modelling frameworks to account for whole-patient factors, ensuring that models reflect the complex, multifactorial nature of hearing outcomes in real-world clinical populations.

Given advances in technology, CI recipients will expect more personalised, evidence-based approaches in the future [42] that are responsive to their specific hearing needs and the subtleties of their individual auditory systems. Biophysical computational models, integrated into clinical protocols, can help meet these expectations by improving device programming, diagnostics, and intervention strategies. While this holds true on a global scale, the impact of model-based care is expected to be a function of the availability and accessibility of expert clinical care and facilities. In developed countries, care is founded on an in-person, engaged and interactive support model, where computational models can provide incremental gains to an already existing and extensive support system. However, model-based care for CI recipients in developing countries could provide essential gains, such as bridging expert skills and gaps in facilities while lowering the cost and logistical effort to access continued support. Such models, therefore, offer a viable pathway to improve access to specialised care in resource-limited settings, where CI adoption is often hindered by cost and logistical barriers. By leveraging standardised data collection, rigorous validation frameworks, and iterative deployment strategies, biophysical models can bridge the gap between their present primarily research-focused application and clinical practice, ultimately providing access to high-quality care for CI recipients worldwide.

## Data Availability

Not applicable.

## Abbreviations

The following abbreviations are used in this manuscript:

AI	Artificial intelligence
CBCT	Cone-beam computed tomography
CI	Cochlear implant
DR	Dynamic range
ECAP	Electrically evoked compound action potential
FNS	Facial nerve stimulation
FOX	Fitting to outcomes eXpert
LMICs	Low- and middle-income countries
NAS	Non-auditory stimulation
WHO	World Health Organisation
